# Innovative opportunities for gene editing technology in crop breeding: from the perspective of literature analysis

**DOI:** 10.3389/fpls.2025.1636024

**Published:** 2025-09-04

**Authors:** Hui Zhang, Ru Yao, Qian Jia, Shijie Qi, Ying He, Jingjuan Zhao, Limin Chuan

**Affiliations:** ^1^ Institute of Data Science and Agricultural Economics, Beijing Academy of Agriculture and Forestry Sciences, Beijing, China; ^2^ Agricultural Information Institute, Chinese Academy of Agricultural Sciences, Beijing, China

**Keywords:** gene editing technology, crop breeding, literature mining, key technology, technology opportunity identification

## Abstract

Gene editing technology is a revolutionary biotechnology that has shown great potential and advantages in crop breeding. Current research has proposed many technical methods and design schemes for gene editing technology in crop breeding. However, summarization and analysis are often based on the research and application of a certain technology, lacking a literature content mining perspective to summarize and analyze the application of gene editing and other technologies in crop breeding. At the same time, there is insufficient identification of future research and innovation opportunities of gene editing technology in crop breeding. This study utilized natural language processing, deep learning, and generative topographic mapping (GTM) to conduct an in-depth analysis of the literature on gene editing technology in crop breeding from the perspective of literature mining. Key technical terms in this field were identified, a literature technical map was constructed, technical blank points were identified, and innovative opportunities for blank technology combinations were analyzed. The results showed that from the literature data from 2020 to 2024, 13 technology combinations were identified. These technical contents cover the multi-technology combination strategy of molecular genetic research, the core technology of gene function research in molecular genetics of biotic and abiotic stresses, the technical means of analyzing the molecular mechanisms of stress resistance, the technical scheme of genetic improvement, etc., which provide support for revealing the potential technological innovation opportunities of gene editing technology in the field of crop breeding. This study can scientifically, objectively, and efficiently identify technological innovation opportunities from the literature. Based on the research results, future research should carry out experimental research and application exploration so as to support the application and technological innovation of gene editing technology in crop breeding.

## 1 Introduction

Crop breeding is an important component of agricultural biotechnology. Through selection, hybridization, and molecular biology, new varieties with excellent traits, such as high yield, disease resistance, stress resistance, and high quality, were bred to meet agricultural production and market demand (Jiang et al., 2023). With the continuous growth of the global population and the increasing impact of climate change, cultivating high-yield, high-quality, and strong stress-resistant crop varieties in a short time has become an urgent problem to be solved (Zainuddin et al., 2024). Traditional crop breeding relies on phenotypic selection, with superior offspring selected based on observed variations in crop phenotypes. This method is highly dependent on the experience of breeding experts and is time-consuming and inefficient, making it difficult to meet the rapidly changing breeding needs (Tyagi et al., 2024). With the continuous innovation and breakthroughs of breeding technology, cutting-edge biotechnologies such as transgenic technology, gene editing technology, double haploid technology, and synthetic apomixis technology have emerged, providing new opportunities for crop breeding.

As a revolutionary biotechnology, gene editing technology has shown great potential and advantages in crop breeding. By precisely modifying specific gene sequences in crop genomes, gene editing technology can achieve specific trait improvements in the precision and efficiency of breeding (Li et al., 2020; Nerkar et al., 2022; Li et al., 2024). With the deepening understanding of crop trait regulation mechanisms by scientists and the development of gene editing tools, precise gene localization, insertion, and knockout of target genes can be achieved, thereby eliminating or reducing certain adverse traits, subsequently enhancing crop disease resistance, insect resistance, and nutritional value. This technology not only shortens the breeding cycle but also reduces the research and development cost. It provides strong technical support for global food security. In addition, gene editing technology can also improve crop varieties according to specific environmental conditions, making them better at adapting to different growing environments, thereby improving the stability and sustainability of agricultural production (Gupta et al., 2024). Therefore, research on the technological innovation of gene editing technology in crop breeding will bring a revolutionary breakthrough for the sustainable development of agriculture. The precise regulation of crop genes can break through the limitations of traditional breeding and also solve global challenges such as food security, climate change, and nutritional health.

In recent years, significant progress has been made in the application research of gene editing technology in crop breeding. Dong (2024) elaborated on the application of mutations in regulatory regions via CRISPR/Cas techniques in crop breeding. Shi et al. (2023) provided an update on the application of promoter editing in crops for increased yield, enhanced tolerance to biotic and abiotic stresses, and improved quality. Yu et al. (2023) analyzed optimization methods for improving prime editing efficiency and their potential in improving crop breeding. Mukhtiar et al. (2025) highlighted CRISPR/Cas and its expanding suite of tools, such as polygenic editing, while delving into its latest innovative technologies, including tissue-specific editing, CRISPR-based gene drives, and epigenetic modifications through dCas9. Song et al. (2025) proposed to use plant prime editing (PPE) for precise plant genome modification, which overcame the limitations of traditional gene editing methods that rely on double-strand breaks and exogenous donor DNA. Liu et al. (2025) focused on three modules that drive precise DNA changes: DNA-targeting modules, effector modules, and control modules, as well as their optimization methods. At the same time, the paper also outlined innovative tools such as optogenetic systems and receptor-integrated systems that enable spatiotemporal control of genome editing expression. Li W. et al. (2025) reviewed the transformative role of large language models (LLMs) in synthetic biology (SynBio) education and research and summarized and analyzed the progress and development potential of LLMs in biomanufacturing. Zhang et al. (2025) established a vector database of over 60,000 research articles for retrieval and enhanced generation and fine-tuned the Llama3-8B model using the language data of 13,993 *Arabidopsis thaliana* phenotypes and 23,323 gene functions to construct a virtual expert PlantGPT for *Arabidopsis* phenotype gene research, providing support for functional genomics research of food crops.

The identification of technology opportunities is a process of technology monitoring and analysis based on bibliometrics and expert opinions (Liu et al., 2023). The analysis methods of technology opportunity are mainly divided into qualitative analysis and quantitative analysis. In terms of qualitative analysis methods, such as the Delphi method and technology roadmap, these methods rely on the knowledge and experience of domain experts. Experts make judgments and evaluations on technical opportunities after investigation and research. This kind of method makes full use of the collective wisdom and experience of domain experts and has been relatively mature through the comprehensive sorting, induction, and analysis of different ideas and views to identify technical opportunities. However, such methods are time-consuming, highly subjective, and easily limited by the breadth and depth of technical experts’ knowledge, resulting in the problem of result deviation. In terms of quantitative analysis methods, many experts and scholars use artificial intelligence methods such as machine learning, network analysis, and link prediction to carry out research on technology opportunity identification. Park and Yoon (2018) used the link prediction method based on citation network to construct potential technological knowledge flows (TKFs) and then extracted the converged technology opportunities by predicting the potential technological knowledge flow between different fields. Cao et al. (2023) adopted semantic analysis and dynamic network analysis methods to capture keyword semantics, mine keyword networks, and realize the potential recombination opportunities of detecting core keywords. Yang et al. (2022) identified technology opportunities for radical inventions (RIs) by measuring the value of technological novelty (VON) of each technology manifested in a patent set and the value of difficulty (VOD) of each research and development (R&D) theme contained in the patent set.

At present, there are many methods to identify technology opportunities. Among them, generative topographic mapping (GTM) is a non-linear hidden- variable model in the field of machine learning, which can realize the non-linear dimensionality reduction of high-dimensional data and is widely used in data analysis and visual analysis (Bishop et al., 1998; Kaneko, 2019). Compared with other technology recognition methods, GTM can maintain the original topological relative relationship of data in high-dimensional space as much as possible and establish a bidirectional mapping between high-dimensional and low-dimensional space. Using the GTM method to draw the technical map, the technical blank points can be visually displayed on the map. At the same time, using the reverse mapping method of GTM can realize the combination of technical words involved in obtaining the blank points in the map. Therefore, the GTM method for technology blank recognition has an excellent visualization effect and can reverse interpret the technology blank. Many scholars have carried out technology opportunity identification based on the GTM method. Zhou and Ban (2023) established a research framework for the identification and evaluation of potential technology opportunities based on GTM and system dynamics (SD) modeling from the perspective of value proposition, further improved the accuracy of technology opportunity identification, and provided theoretical and practical support for enterprises to optimize the layout of technology directions with the value orientation of technology. The research is applied in the field of new energy vehicles. Li et al. (2023) first used the GTM patent map to identify technical gaps and reverse interpret them, then clustered scientific literature to obtain scientific knowledge topics, and finally evaluated the similarity between potential technical opportunities and scientific topics through the cosine similarity value to screen out scientific and feasible technological innovation opportunities. This research is applied in the field of medical equipment. Wang et al. (2022) proposed an automated technology opportunity discovery method combining subject–action–object (SAO) and GTM. The automated method focusing on semantic information helps to understand technology opportunities and improve the accuracy of discovery. This research is applied in the field of coal bed methane extraction technology.

In summary, the current research has proposed many technical methods and design schemes for gene editing technology in crop breeding. However, it is often based on the research and application of a certain technology, lacking a literature content mining perspective to summarize and analyze the application of gene editing and other technologies in crop breeding. There is also insufficient identification of future research and innovation opportunities of gene editing technology in crop breeding. At the same time, when using a variety of data mining and analysis methods to identify and analyze technology opportunities, many scholars have put forward innovative and practical technical methods. However, there is also the possibility of omitting technical points by analyzing subject words and keywords as technical elements. In addition, the identification of technological innovation opportunities is very important for technological research and development and breakthroughs, but there is no research achievement on the identification of technology opportunities of gene editing technology in crop breeding in the public literature.

Therefore, based on the research literature dataset of gene editing technology in the field of crop breeding, this paper used spaCy, SciBERT, and GTM methods to identify potential innovation opportunities for gene editing technology and conducted an in-depth analysis of technological innovation opportunities to provide support for the future technological innovation and development of gene editing technology in crop breeding. This study used spaCy’s named entity recognition (NER) method to identify key technical words in literature titles and abstracts and chose to add the SciBERT pre-trained model to the spaCy pipeline for model training. SciBERT (Beltagy et al., 2019) is a BERT model that uses a large corpus of scientific publications, including a total of 1.14 million samples of papers in biomedicine (82%) and computer science (18%), for unsupervised pre-training, making it more suitable for natural language processing tasks on literature data. Based on the training dataset, the NER model fused with Transformer was constructed and trained.

Traditional expert judgment methods are subjective, one-sided, and easily influenced by the breadth and depth of expert knowledge. In addition, compared with the various current large language models, the advantages of using SciBERT for named entity recognition are as follows:

- Professional advantages in semantic understanding. The big language model is an autoregressive generative model, whose training objective is to predict the probability distribution of the next word based on a given preceding text. Its training method is unidirectional, making it particularly powerful in generating coherent text. However, its understanding of context is relatively one-sided compared to SciBERT. The core idea of SciBERT is to pre- train the encoder through bidirectional context so that semantic information can be obtained from the bidirectional context, making its understanding of text more comprehensive.
- Professional advantages in the research field. Although the big language model has extensive knowledge, there is a problem of insufficient recognition accuracy when performing specialized terminology recognition due to the use of models trained on general corpora. SciBERT utilizes a pre- trained BERT model based on a large library of scientific publications, which provides a more accurate understanding of professional terms appearing in literature and is suitable for identifying named entities.
- Advantages of structured information extraction. After extracting structured information, the big language model requires additional standardization processing, which is less efficient compared to using the SpaCy+SciBERT method. The SpaCy+SciBERT method can combine multiple rules for precise extraction while providing a standardized NER pipeline to output structured entity labels for subsequent data analysis.
- Advantages in efficiency and cost. The fine-tuning cost of large language models is relatively high, requiring a large amount of computing power for inference, and there is also an illusion effect.

The aim of this study was to apply natural language processing technology, deep learning, and GTM methods to identify potential innovation opportunities for gene editing technology and conduct an in-depth analysis of technological innovation opportunities. By integrating multiple data mining and technology opportunity identification methods, potential technological discoveries can be provided for the development of new editing tools, precise regulation techniques, gene delivery mechanisms, off- target effects, and other directions. This has high application prospects for improving the efficiency and accuracy of genome editing technology, developing artificial intelligence (AI)-powered breeding tools, and other directions.

## 2 Materials and methods

### 2.1 Data sources

The literature data for this study were retrieved from the Web of Science Core Collection of the Science Citation Index Expanded database, and the search time was January 3, 2025. Subject keywords were used to retrieve a literature dataset of gene editing technology in crop breeding. Considering that the literature before 2020 may have deficiencies in novelty, in our study, the publication date was set from January 1, 2020, to December 31, 2024, and the literature types were limited to research papers and reviews. To ensure the quality of the literature, this study manually browsed through the titles and abstracts of the literature to determine whether the article was relevant to the selected target research topic in order to eliminate irrelevant literature. Finally, this study obtained a basic dataset of 17,234 literature, including 15,880 papers and 1,354 reviews.

### 2.2 Recognition of key technical words

spaCy’s NER method was used to identify key technical words in literature titles and abstracts. The spaCy is a natural language processing library written based on Python. Using spaCy’s named entity recognition method, entities with specific meanings can be extracted from unstructured text. The predefined NER tags of spaCy do not involve specific types of key technical entities, so the custom NER model was trained through spaCy to recognize key technical words of crop breeding.

#### 2.2.1 Dataset construction

To ensure the effectiveness and accuracy of model training, a high-quality and standardized training dataset was constructed. Based on the literature title and abstract texts obtained from previous searches, 1,000 pieces of literature were randomly selected as the training dataset source. Key technical words that appeared in the dataset were annotated manually. Finally, a dataset that met the training requirements was obtained.

#### 2.2.2 Recognition model training

**Figure 1:**
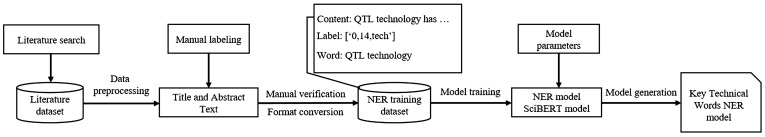
Construction process of key technology word recognition model.

spaCy allows using Transformer models (such as BERT, GPT-2, and XLNet) for natural language processing tasks. Therefore, combining the powerful context understanding capabilities of Transformer models for NER model training can produce better recognition effects. The SciBERT pre-trained model was added to the spaCy pipeline for model training. Based on the training dataset, the NER model fused with Transformer was constructed and trained. After multiple rounds of parameter tuning, a NER model that met the key technical word recognition requirements for crop breeding was finally obtained. The model construction process is shown in **Figure 1**.

### 2.3 Recognition of technical blank points

Technology map was used to identify the blank of gene editing technology. Technology map is composed of technical data information, and the original high-dimensional data space was mapped to a low-dimensional regular grid by an algorithm. The blank points displayed in the technical map represent the lack of corresponding technical combinations for the coordinate point, which are called technical blank points. By systematically analyzing and interpreting these technical blank points, potential opportunities for technological innovation can be effectively identified, providing new breakthrough directions for technological development.

**Figure 2:**
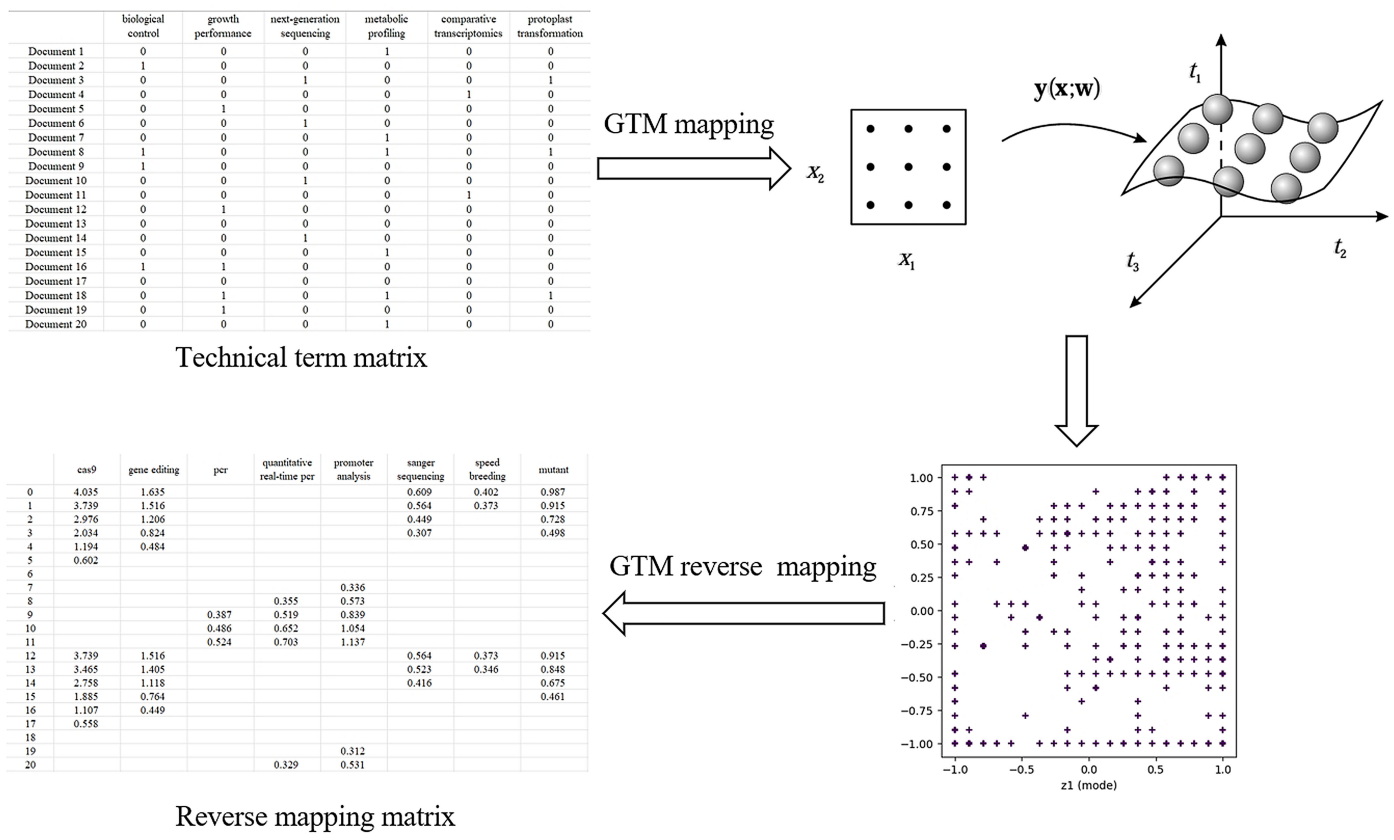
Process diagram for technical blank identification flowchart.

The GTM was used to draw technical maps. It helps to understand and analyze complex datasets by mapping high-dimensional data to low-dimensional latent space. The flowchart of technical blank identification is shown in **Figure 2**.

**Figure 3:**
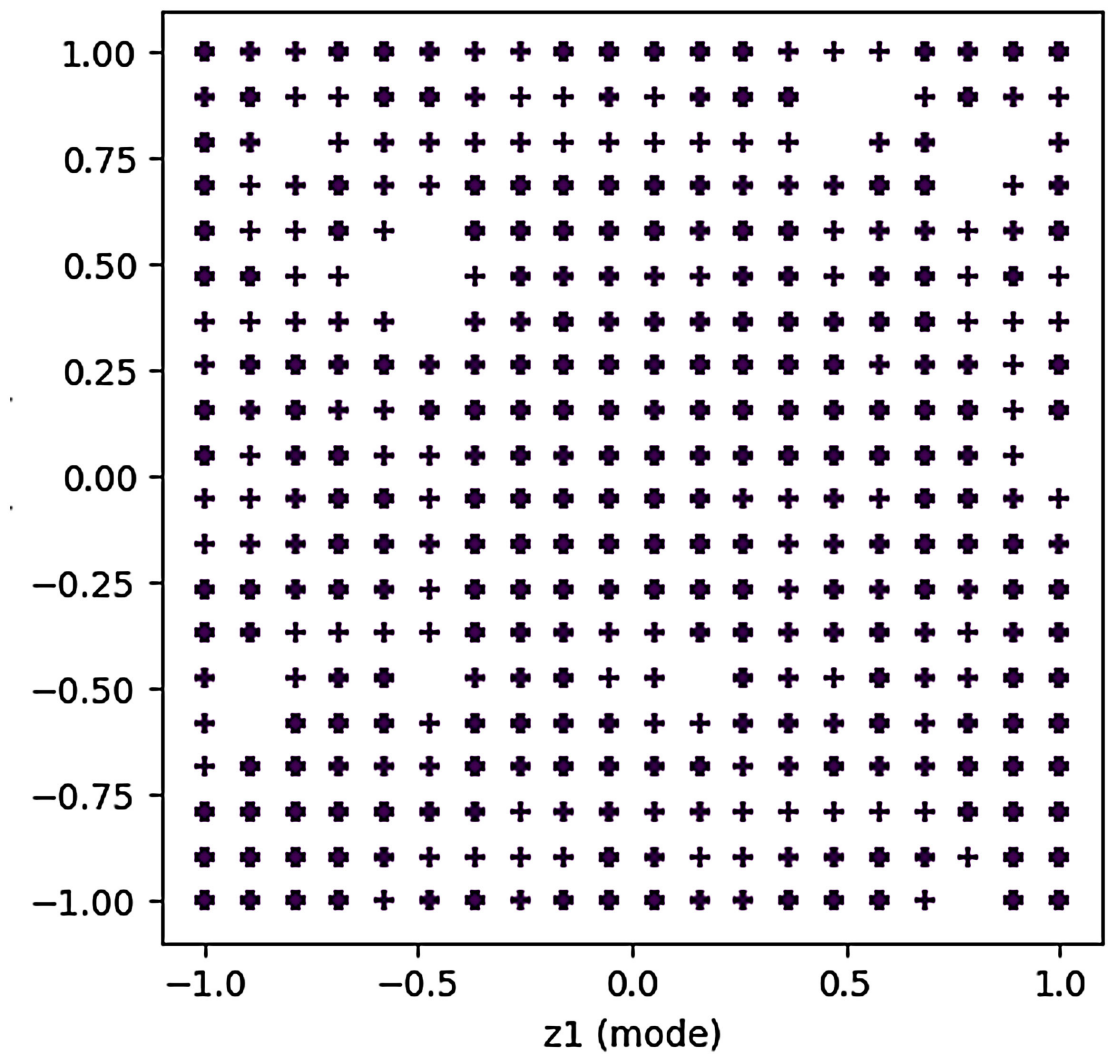
Example of technology map generated based on GTM. GTM, generative topographic mapping.

Based on the key technical word recognition model constructed in the early stage, the key technical words were recognized in the breeding literature data, and the key technical words appearing in the literature were obtained. After data deduplication and synonym merging, key technical words from the literature were selected and confirmed by domain experts. According to the literature title and abstract text, the key technical word representation vector of the binary representation was constructed. The construction method is as follows. When the title or abstract text of a paper contains the identified key technical words, the corresponding element value in the technical word vector is 1, and 0 otherwise, and finally, the technical word matrix of the paper data is formed. The technical word matrix is shown in **Table 1**. The constructed multi-dimensional technical feature word matrix is imported into the GTM model, and the multi-dimensional vector is reduced to two-dimensional space, in which each technical data point is mapped to a two-dimensional plane. Specific points in the map represent existing technologies in the technical field, and blank areas in the technical field are potential technology opportunities, as shown in **Figure 3**. On this basis, the potential technology opportunities are reversely mapped back to the multi-dimensional data blank through GTM reverse mapping, as shown in **Table 2**. By setting the threshold to screen technical words and combining it with the analysis of technical words by experts, the interpretation of potential technical opportunities is completed.

**Table 1:** Example of the binary representation matrix of key technology terms.

Words Doc ID	Biological control	Growth performance	Next-generation sequencing	…	Metabolic profiling
1	0	0	0	…	1
2	1	0	0	…	0
3	0	0	1	…	0
4	0	0	0	…	0
5	0	1	0	…	0
6	0	0	1	…	0
7	0	0	0	…	1
8	1	0	0	…	1
9	1	0	0	…	0
10	0	0	1	…	0
11	0	0	0	…	0
…	…	…	…	…	0
17,234	0	0	0	…	0

“Words” represents the terms (a total of 468 columns), and “Doc ID” represents the identification number of each article (a total of 17,234 rows). If the title or abstract text of the paper contains words, the corresponding element value of the words is 1, and 0 otherwise.

**Table 2:** Example of results after reverse mapping based on the GTM model.

Index Map location	1	2	3	…	N
1	−0.126	0.137	0.129	…	−0.084
2	−0.144	0.146	0.155	…	−0.215
3	−0.157	0.144	0.176	…	−0.386
4	−0.167	0.141	0.187	…	−0.534
5	−0.173	0.138	0.179	…	−0.585
6	−0.175	0.138	0.148	…	−0.518
7	−0.174	0.141	0.104	…	−0.387
8	−0.171	0.146	0.067	…	−0.276
9	−0.160	0.150	0.051	…	−0.243
10	−0.140	0.153	0.057	…	−0.293
11	−0.117	0.156	0.075	…	−0.388
…	…	…	…	…	−0.468
M	−0.095	0.155	0.077	…	−0.413

“Index” represents the word number (N technical words in total), and “Map location” represents the point number on the technical map (M points in total; if the map size is 20 * 20, then M = 400).

GTM, generative topographic mapping.

## 3 Results

### 3.1 Recognition results of key technical words

#### 3.1.1 Data labeling

First, we specified that the marked contents are words, phrases, abbreviations of terms, etc., indicating technology. Second, we invited two experts in the field to carry out the annotation work in the way of independent annotation. Finally, we determined the final labeled entity training set by negotiating the inconsistent labeling content. In the process of annotation, we required domain experts to read and analyze the title and abstract text and mark the technical words appearing in the text. At the same time, we used natural language processing tools to extract the sentence and location information of the marked words and convert it into a format consistent with the training data. Finally, through data cleaning, manual verification, and other operations, we obtained 757 labeled data points that met the training data requirements. An example of the labeling result is shown in **Table 3**, where Content is the original sentence, Label is the starting position information of the technical word and the technical word tag, and Tech_word is the technical word.

**Table 3:** Example of key technical word tagging results.

ID	Content	Label	Tech_word
1	High-throughput sequencing results suggested that the bacterial diversity in CS rumen fermentation and fungal diversity and richness in WS rumen fermentation were promoted with FW as a co-substrate.	[‘0,26,tech’]	[‘High-throughput sequencing’]
2	Amplification sub-sequencing techniques were used to compare soil community structures among the systems and identify significant positive and negative fertility factors.	[‘0,38,tech’]	[‘Amplification sub-sequencing techniques’]
3	Genetic mapping and genome assembly revealed three flowering- promoting factor 1 (FPF1) genes located within the BR mapping interval, and a complete coding sequence deletion of the telomere proximal FPF1 (Solyc01g066980) was found in the br allele but not in BR.	[‘0,15,tech’, ‘20,35,tech’]	[‘genome assembly’, ‘Genetic mapping’]
4	A genetic linkage map was constructed using single-nucleotide polymorphism markers generated by genotyping-by-sequencing.	[‘2,21,tech’]	[‘genetic linkage map’]
5	Syntenic analysis verified *Brassica* genome triplication, and whole- genome duplication likely contributed to the expansion of the OFP gene family.	[‘0,17,tech’]	[‘Syntenic analysis’]

“Content” is the original sentence, “Label” is the starting position information of the technical word and the technical word tag (tech), and “Tech_word” is the marked technical word.

#### 3.1.2 Technical word recognition

A named entity recognition model was constructed based on NER+SciBERT, and the model was trained with labeled data to generate a technical word recognition model consistent with this study. The optimization results of model training parameters are shown in **Table 4**. Using the key technical word recognition model, the key technical words of literature titles and abstract texts in the literature dataset in the field of crop breeding were recognized, and candidate key technical words were obtained. After data deduplication and synonym combination, 468 technical words were obtained. The BERT, BioBERT, and SciBERT models were used to compare the performance, and the precision, recall, and F1-score of the recognition task were evaluated. The experimental results are shown in **Table 5**. Finally, the performance of the SciBERT model for the annotation task in this study was verified.

**Table 4:** NER-SciBERT training parameter setting.

Parameter name	Parameter settings
learn_rate	1e−4
drop	0.5
iterations	500
batch_size_start	4
batch_size_stop	32
batch_size_growth_factor	1.001

“learn_rate” represents the learning rate. “drop” represents dropout rate, which is a regularization technique used in neural network training. “iterations” represents the number of iterations. “batch_size_start” represents the initial batch size. “batch_size_stop” represents the final batch size. “batch_size_growth_factor” represents the batch growth rate.

**Table 5:** Based on the experimental results of BERT, BioBERT, and SciBERT models.

Model name	Precision	Recall	F1-score
BERT	0.506	0.494	0.500
BioBERT	0.493	0.430	0.460
SciBERT	0.553	0.658	0.601

### 3.2 Technology opportunity analysis

#### 3.2.1 Technical blank identification results

Based on the key technical words identified above, and combined with the literature information, the technical word matrix of literature data was constructed, and the multi-dimensional technical feature word matrix was input into the GTM model. The selection of GTM model parameters affected the visualization effect of the technical map. Through multiple parameter adjustments, the display effect of the technical map and the results of reverse mapping were checked, and the best GTM model parameters of this study were finally obtained. The specific parameters are shown in **Table 6**. Map size controls the resolution of the latent grid, affecting model complexity and detail capture. Shape of Radial Basis Function (RBF) centers determines the layout of basis functions, influencing mapping uniformity. Variance of RBFs adjusts the width of basis functions, balancing smoothness and overfitting risk. Lambda in EM algorithm regularizes model weights to trade off complexity and generalization. Iterations sets the maximum EM steps, impacting convergence and computational cost.

**Table 6:** Optimal parameters for generative topographic mapping (GTM) model.

Parameter name	Parameter settings
Map size	20 * 20
Shape of RBF centers	6,6
Variance of RBFs	0.05
Lambda in EM algorithm	0.01
Iterations	5

Map size: the shape of the latent space grid. Shape of RBF centers: the shape of the radial basis function grid. Variance of RBFs: the width of the radial basis functions. Lambda in EM algorithm: the regularization coefficient. Iterations: the number of iterations.

**Figure 4:**
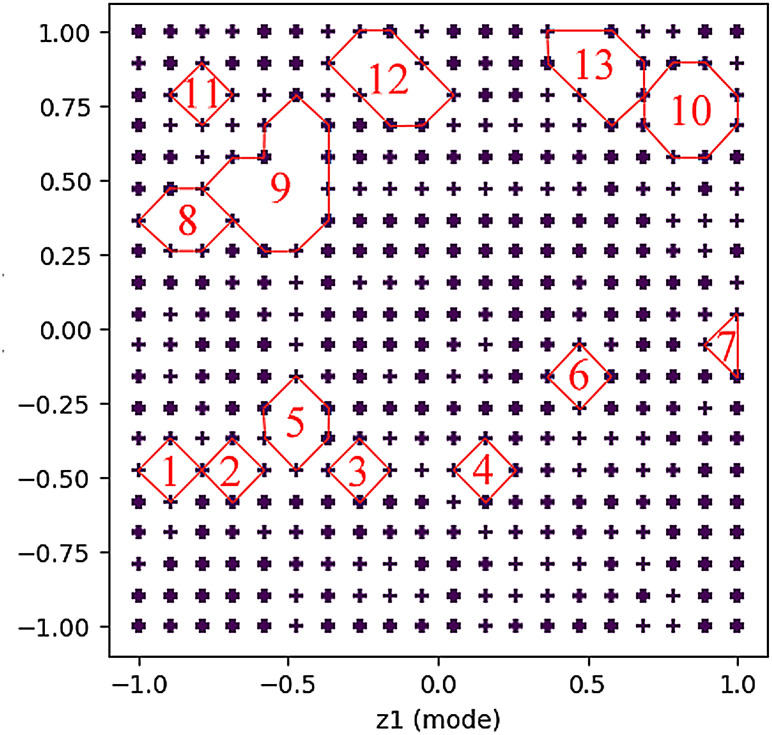
Technical map results generated based on GTM. A total of 13 groups of technology combinations are marked. GTM, generative topographic mapping.

On this basis, we drew the technical map using the GTM model, as shown in **Figure 4**. There are 13 blank spots in the figure, and the specific positions are shown in **Table 7**. The blank spots are marked with Arabic numerals 1–13 in the order from left to right and from bottom to top. Blank spot location represents the location number on the map corresponding to the blank spot. For the screening of technical terms in GTM reverse mapping results, we referred to the threshold set in the experiment in the relevant literature (Zhou and Ban, 2023). At the same time, we focused on the technical words in the GTM reverse mapping results and finally determined that the threshold setting of 0.3 is more reasonable. We analyzed the blank spots, and we screened the technical words in the blank spots by setting the threshold value to 0.3. The screening results are shown in **Table 8**. Among them, each column represents a technical word, and each row represents the position of technical map points. Therefore, we extracted the technical combination words included in each technical blank point in combination with the blank points in **Table 7**. In order to facilitate intuitive viewing and analysis of technical combinations, we screened technical words, among which **Table 9** shows the technical words that appeared commonly in technical combinations, and **Table 10** shows the technical words that did not appear commonly in technical combinations. Domain experts can realize the interpretation and analysis of technical combinations by comprehensively analyzing technical words in the two tables.

**Table 7:** Location information corresponding to blank points in the technical map.

Blank spot number	Blank spot location
1	102
2	104
3	108
4	112
5	126; 146
6	175
7	199
8	262; 263
9	265; 266; 284; 285; 286; 306; 326
10	338; 339; 358; 359
11	343
12	349; 350; 368; 369
13	356; 375; 376; 395

“Blank spot number” is the number of the blank spot, and “Blank spot location” represents the location number on the map corresponding to the blank spot.

**Table 8:** Threshold screening based on the reverse mapping results of the GTM model (partial results of the first 10 points), and the results greater than the threshold of 0.3 are retained.

Words Map location	Transgene	Functional complementation	Genomic selection	…	Genetic mapping
1	0.504	–	–	…	–
2	0.433	–	–	…	–
3	–	–	–	…	–
4	–	–	–	…	–
5	–	–	–	…	0.330
6	–	0.304	–	…	0.591
7	–	0.306	0.593	…	0.910
8	–	0.304	1.125	…	1.134
9	–	–	0.948	…	1.113
10	–	–	–	–	0.834

“Words” stands for technical words (468 words in total), and “Map location” denotes the point number on the technical map (400 points in total according to the experimental results).

GTM, generative topographic mapping.

**Table 9:** Common technical terms in technical combinations.

Number	Technical words
1	acyl-CoA-binding protein; biotic stress; BSA-seq; bulk; cadmium stress; Cas9; chemical; chitinase; cloned; cold resistance; cold treatment; consistent; Crispr; DNA sequence; field experiment; field test; fluorescent protein; functional assay; genetic network; genome editing; genome-wide analysis; heterologous expression; high temperature; histochemical analysis; in-depth analysis; indeed; industrial production; knockdown; linkage analysis; metabolic engineering; monomeric; multiple stress; mutation; overexpression; protoplast system; quantitative polymerase chain reaction; recombinant; repression; sequence analysis; stem; tertiary structure; thuringiensis; verticillium; vitro

**Table 10:** Non-common technical terms in technical combinations.

Blank spot	Technical words
1	transgene; functional complementation; genetically modified; mutant; mediated; various stress; complement; multiplex editing; knockout; Spodoptera frugiperda; functional analysis; Southern blot; histochemical staining
2	transgene; functional complementation; large-scale; mutant; ethyl methane sulfonate; homologous recombination; various stress; complement; genetic analysis; inoculation; meiotic recombination; cross-resistance; evaluation; Southern blot
3	genetic resistance; mutant; various stress; inoculation; comparative study; targeted editing; endosperm-specific; biotechnological tool; endosperm; evaluation; enzyme assay
4	next-generation sequencing; functional complementation; dissected; KASP; mutant; ethyl methane sulfonate; ChIP-seq; integrated analysis; various stress; complement; chromatin; genetic analysis; inoculation; aligned; targeted editing; genome assembly; RNA-directed; genetic control; cold stress; high-affinity; high-resolution; functional analysis
5	transgene; large-scale; mutant; inoculation; comparative study; targeted editing; cross-resistance; evaluation; foliar application
6	methylation status; functional complementation; mutant; mediated; plant response; differential expression; WGCNA; inoculation; comparative study; bisulfite sequencing; gene network; targeted editing; CREs; expansions; cold stress; high-affinity; functional analysis; gene expression; meta-analysis; histochemical staining
7	functional complementation; plant response; xylem sap; HSFs; sequence alignment; various stress; differential expression; complement; xylem; secondary structure; inoculation; targeted editing; CREs; positive selection; tandem; cold stress; functional analysis; RT-qPCR; stress; histochemical staining; high expression; subcellular localization
8	functional complementation; confocal microscopy; various stress; complement; tissue culture; inoculation; bacterium; microorganism; targeted editing; electroporation; Azospirillum; cut; evaluation; growth performance; mutant; cross-resistance; high-affinity; colonization
9	growth performance; functional complementation; confocal microscopy; mutant; various stress; complement; inoculation; targeted editing; inhibiting; cross-resistance; arbuscular mycorrhizal; high-affinity; enzyme assay; colonization; stress; EPS; histochemical staining; fluorescence microscopy; mediated; functional analysis; Nilaparvata lugens
10	functional complementation; mutant; osmotic stress; mediated; plant response; complement; inoculation; aligned; real-time PCR; high salinity; targeted editing; cross-resistance; proteomic analysis; cold stress; high-affinity; transactivation; functional analysis; histochemical staining; enzyme assay; yeast one-hybrid; tandem; secondary metabolism; various stress; transcript; virus-induced gene silencing; silencing; gene silencing; genetic mapping; CKX
11	functional complementation; confocal microscopy; mutant; mediated; various stress; complement; stress; inoculation; Nilaparvata lugens; targeted editing; cross-resistance; arbuscular mycorrhizal; high-affinity; functional analysis; evaluation; histochemical staining; colonization
12	functional complementation; mutant; mediated; plant response; various stress; inoculation; targeted editing; inhibiting; ethylene production; cold stress; high-affinity; transactivation; carotenoid content; functional analysis; fluorescence microscopy; stem elongation; enzyme assay; ChIP-qPCR; stress; yeast one-hybrid; histochemical staining; transcript; tandem
13	functional complementation; mutant; osmotic stress; mediated; plant response; various stress; complement; transcript; inoculation; high salinity; silencing; targeted editing; cold stress; high-affinity; transactivation; functional analysis; PEG-mediated; gene silencing; histochemical staining; enzyme assay; race; yeast one-hybrid; that silencing; HIGS; host-induced gene silencing; transgene; cut; luciferase

#### 3.2.2 Technical combination analysis

##### 3.2.2.1 Application of CRISPR/Cas9 technology in the precise regulation of agricultural biology

The first technology combination complements molecular and genetic research. By comprehensively applying technical methods such as transgene, mutant analysis, and functional complementarity analysis, the function of genes and their mechanism of action in organisms can be more comprehensively revealed. Understanding the potential uses of related technologies helps to develop new strategies for targeting key functional genes. Related research: “PEG-Delivered CRISPR-Cas9 Ribonucleoproteins System for Gene-Editing Screening of Maize Protoplasts” (Sant’Ana et al., 2020) and “CRISPR/Cas9 mediated editing of *pheromone biosynthesis activating neuropeptide* (*PBAN*) gene disrupts mating in the Fall armyworm, *Spodoptera frugiperda*” (Ashok et al., 2023).

##### 3.2.2.2 Collaboration between gene editing and transformation technology promotes research on rice breeding

The second technology combination covers the core technologies of gene manipulation (transgenic and homologous recombination), mutant library construction [ethyl methane sulfonate (EMS) mutagenesis and large-scale screening], functional verification (complementary experiments and Southern blotting), and multi-dimensional evaluation (stress resistance and safety), which together constitute the core tool chain of stress resistance and disease resistance molecular genetics research. Related research: “Concurrent Disruption of Genetic Interference and Increase of Genetic Recombination Frequency in Hybrid Rice Using CRISPR/Cas9” (Liu et al., 2021) and “*Agrobacterium*-Mediated Genetic Transformation of Wild *Oryza* Species Using Immature Embryos” (Shimizu-Sato et al., 2020).

##### 3.2.2.3 Research on the application of biotechnology in crop improvement

The third technology combination forms a research chain of targeted editing to create mutants, inoculating pathogens or applying stress, and evaluating resistance through enzyme activity and phenotype; and to compare and analyze to optimize editing strategies, providing new research strategies for analyzing molecular biology research on disease resistance and abiotic stress. Related research: “Overexpression of Nepenthesin *HvNEP-1* in Barley Endosperm Reduces Fusarium Head Blight and Mycotoxin Accumulation” (Bekalu et al., 2020) and “CRISPR/Cas9-mediated editing of phytoene desaturase (*PDS*) gene in an important staple crop, potato” (Siddappa et al., 2023).

##### 3.2.2.4 Technical innovation of crop physiological metabolism and quality improvement

The fourth technology combination forms a complete research chain from gene discovery [next-generation sequencing (NGS) and mutant screening] to functional verification (complementation and editing) and then to mechanism analysis [Chromatin immunoprecipitation followed by sequencing (ChIP-seq) and interaction analysis), providing a new technical method for analyzing stress- resistant molecular genetic biology research. Future research trends can focus on the combination of multiple technologies (such as single-cell sequencing combined with spatial transcriptome) and high-throughput automated analysis. Related research: “Mass screening of rice mutant populations at low CO2 for identification of lowered photorespiration and respiration rates” (Mubarak et al., 2023) and “The molecular mechanism of polyphenol oxidase 1 and the genetic improvement of wheat polyphenol oxidase activity” (Zhai et al., 2023).

##### 3.2.2.5 Research on precision breeding strategies and tools

The fifth technology combination achieves the cultivation of new stress-resistant varieties by combining targeted editing and resistance assessment in agriculture through precise genetic manipulation, systematic phenotypic analysis, and multi-level evaluation. The combination of technologies provides experimental protocols to promote molecular mechanism analysis and genetic improvement. Related research: “Loss of Function of OsFBX267 and OsGA20ox2 in Rice Promotes Early Maturing and Semi-Dwarfism in γ-Irradiated IWP and Genome-Edited Pusa Basmati-1” (Andrew-Peter-Leon et al., 2021) and “Genome-scale targeted mutagenesis in *Brassica napus* using a pooled CRISPR library” (He et al., 2023). By optimizing codon, promoter, and editing conditions, the prime editors were adapted for use in plants, thereby achieving point mutations, insertions, and deletions in rice and wheat protoplasts (Lin et al., 2020).

##### 3.2.2.6 Analysis and editing strategy of gene regulatory network for crop traits

The sixth technology combination forms a complete research chain to study the role and regulatory mechanism of key genes from epigenetics (methylation status analysis using bisulfite sequencing and differential expression using RNA-seq) to transcriptional regulation and then to functional verification. Future research can further integrate multi-omics data and spatial–temporal dynamic analysis to improve the accuracy of complex traits. Related research: “Understanding the regulatory relationship of abscisic acid and bZIP transcription factors towards amylose biosynthesis in wheat” (Kumar et al., 2021) and “CRISPR-Cas9 mediated OsMIR168a knockout reveals its pleiotropy in rice” (Zhou et al., 2021).

##### 3.2.2.7 Research on the identification strategy of key genes for crop traits

The seventh technical combination clarifies the direct role of genes through functional verification (such as complementation and editing). Expression and localization techniques reveal spatiotemporal dynamics. Stress response analysis links gene function and phenotype. Evolutionary and structural analysis resolves the molecular basis of functional differentiation. This combination provides abundant technical means for the molecular mechanism of plant stress response and stress resistance. Related research: “The rice annexin gene *OsAnn5* is involved in cold stress tolerance at the seedling stage” (Que et al., 2023) and “*BnaC09.tfl1* controls determinate inflorescence trait in *Brassica napus*” (Zhao et al., 2024).

##### 3.2.2.8 The complete chain of plant– microbe interaction research

The eighth technology combination forms a complete research chain from gene manipulation (targeted editing and electroporation) to functional verification (functional complementation and mutant analysis) and then to phenotypic analysis (confocal imaging and stress response evaluation). This combination provides a core technology for analyzing the molecular and genetic mechanisms of microbial interaction and resistance mechanisms. Related research: “Expression and function of the *cdgD* gene, encoding a CHASE-PAS-DGC-EAL domain protein, in *Azospirillum brasilense*” (Cruz-Pérez et al., 2021) and “Diversity and functional characterization of endophytic Methylobacterium isolated from banana cultivars of South India and its impact on early growth of tissue culture banana plantlets” (Senthilkumar et al., 2021).

##### 3.2.2.9 Regulatory strategies for plant disease resistance

The ninth technology combination is mainly used in pathogen or pest–host interaction research, creating mutant materials through targeted editing, and revealing regulatory functions through resistance identification, enzyme activity measurement, and confocal microscopy. This combination of technologies constitutes a complete research chain from phenotypic observation to molecular mechanism analysis and provides a new strategy for the combination of multiple technologies in molecular genetic research. Related research: “Influence of Enhanced Synthesis of Exopolysaccharides in *Rhizobium ruizarguesonis* and Overproduction of Plant Receptor to these Compounds on Colonizing Activity of Rhizobia in Legume and Non-Legume Plants and Plant Resistance to Phytopathogenic Fungi” (Kantsurova et al., 2024) and “Functional Analysis of *BcSNX3* in Regulating Resistance to Turnip Mosaic Virus (TuMV) by Autophagy in Pak-choi (*Brassica campestris* ssp. *chinensis*)” (Zhang et al., 2022).

##### 3.2.2.10 Research on crop stress resistance and virus functional genomics

The 10th technology combination systematically reveals the technical system of the role of genes in the stress resistance pathway and promotes the analysis from single gene function to complex regulatory network through collaborative application, especially in the fields of plant stress biology and metabolic engineering. Related research: “Transcription factor TabHLH49 positively regulates dehydrin *WZY2* gene expression and enhances drought stress tolerance in wheat” (Liu et al., 2020) and “Long fragment circular efficient PCR (LC-PCR): an integrated technology that modifies large plasmid constructs through site-directed gene insertion, deletion, and mutation, without the need for restriction or ligation” (Jailani et al., 2023).

##### 3.2.2.11 Analysis of pest drug resistance mechanisms and visual detection of symbiotic fungi

The 11th technology combination combines targeted editing and resistance assessment to analyze the cross-resistance mechanism of its drug resistance genes. It can also be used for plant mycorrhizal symbioses, such as dynamic observation of mycelial colonization process by confocal microscopy and histochemical staining. Technology combination provides a new strategy for multi-technology combination in molecular genetic research. Related research: “New insights into chlorantraniliprole metabolic resistance mechanisms mediated by the striped rice borer cytochrome P450 monooxygenases: A case study of metabolic differences” (Xu et al., 2024) and “Anthocyanin pigmentation as a quantitative visual marker for arbuscular mycorrhizal fungal colonization of Medicago truncatula roots” (Kumar et al., 2022).

##### 3.2.2.12 Research on crop development and stress response

The 12th technology combination jointly promotes research progress in gene function analysis, stress response mechanism, metabolic regulation network, and other fields through multi-level and multi-angle research and provides a complete research chain for analyzing the molecular genetic biology of disease resistance and abiotic stress. Related research: “Overexpression of SlGSNOR impairs *in vitro* shoot proliferation and developmental architecture in tomato but confers enhanced disease resistance” (Rasool et al., 2021) and “Excess iron accumulation affects maize endosperm development by inhibiting starch synthesis and inducing DNA damage” (Zang et al., 2024).

##### 3.2.2.13 Research on the mechanism of crop disease resistance

The 13th technology combination plays an important role in analyzing gene function, regulatory mechanisms, and plant response to stress. Each technology has its own unique role and jointly promotes the progress of plant immunity regulation, pathogenic mechanism, crop improvement, and plant biology. Related research: “Wheat Leaf Rust Fungus Effector Protein Pt1641 Is Avirulent to TcLr1” (Chang et al., 2024) and “Host-induced gene silencing of the Verticillium dahliae thiamine transporter protein gene (VdThit) confers resistance to Verticillium wilt in cotton” (Wang et al., 2024).

#### 3.2.3 Technology opportunity assessment

In order to effectively validate our research method, domain experts were invited to analyze 13 sets of technology combinations and rate them based on three dimensions: scientificity, feasibility, and application prospects. On the basis of the score, the comprehensive score of the evaluation index was calculated by summing the evaluation indexes. The objective evaluation of the innovation of the technology combination was ultimately achieved, and the effectiveness of this method was verified.

The technology portfolio evaluation index is described as follows: evaluate the scientificity of the technology portfolio, that is, whether it follows scientific principles and is supported by theory or empirical evidence. Evaluate the feasibility of the technology combination, that is, whether it can actually be applied or realized under the existing conditions. Evaluate the application prospects of the technology portfolio, that is, the potential value in solving practical problems in the field or promoting the development of the field.

Experts in the field need to score 13 sets of technology combinations independently from 1 (poor) to 5 (excellent). The scoring results of technology combinations are shown in **Table 11**. The final score of the technical combination was calculated by scoring 13 groups of technical combinations by experts. From the scoring results, it can be concluded that technology combinations 4, 6, and 10 have more potential for technological innovation. Therefore, researchers can focus on and analyze the application of these technology combinations, find the experimental innovation path, and explore the innovation of technology integration.

**Table 11:** Scoring results of technology combination based on domain experts.

Blank spot number	Scientificity	Feasibility	Application potential	Total score
1	4	4	5	13
2	4	5	5	14
3	3	5	4	12
4	5	5	5	15
5	5	5	4	14
6	5	5	5	15
7	4	5	5	14
8	4	5	4	13
9	4	5	4	13
10	5	5	5	15
11	4	5	4	13
12	4	5	4	13
13	4	5	5	14

Evaluate from three dimensions: scientificity, feasibility, and application potential.

## 4 Discussion

### 4.1 Theoretical implications

The application of gene editing technology in crop breeding is developing rapidly, but it still faces challenges in specificity, precision, delivery efficiency, and safety (Alariqi et al., 2025). Strengthening the integration of gene editing with other technologies (including high-throughput phenotypic analysis, genomic selection, and rapid breeding) is necessary to further promote its widespread application in agriculture (Atia et al., 2024). This study deepened and expanded the application research of gene editing technology in the field of crop breeding by applying new methods and technologies. Through the analysis method of literature mining, the potential research opportunities of gene editing technology in crop breeding were revealed, which provides new insights into and supplements to the existing knowledge system. At the same time, by objectively and scientifically extracting potential technology combination innovation opportunities from massive literature data, it breaks through the limitations of traditional expert experience and provides a data-driven decision-making basis for the future research direction of gene editing technology in the field of crop breeding. The method formed in this study can be extended to other technical fields, providing practical support for the innovation of related theories and technical methods.

### 4.2 Practical implications

Currently, relevant scholars are conducting research on core breeding technology identification, gene discovery, and utilization based on bibliometrics, Latent Dirichlet Allocation (LDA) models, network analysis, knowledge graphs, and other methods (Jia et al., 2025; Zheng, 2025; Sahu et al., 2023), providing support for planning breeding technology research and development directions. At the same time, by developing an automated tool for extracting gene–phenotype associations from literature, they explored the complex correlations between genes and phenotypes in crops, fully exploiting and utilizing data for modern molecular breeding (Gao et al., 2024). Research on innovative opportunities in the research and development direction from the perspective of literature content mining is gradually emerging. This study systematically analyzed the published literature information of gene editing technology in the field of crop breeding by integrating various text mining methods (such as natural language processing, deep learning, and generative topology mapping) and deeply mined and identified potential technological innovation opportunities, forming a set of methodological frameworks for identifying key technologies and technology combinations. This method provides scientific research institutions and enterprises with accurate decision support for R&D direction, helps them optimize scientific research layout, and reduce trial-and-error costs. At the same time, it provides decision-making reference for the improvement of the development policy system, the scientific deployment of special plans, and the investment of R&D funds. In the future, industrial data (such as patents and market reports) can be further combined to build a more comprehensive technology evaluation system to maximize the potential of gene editing technology in crop breeding.

It is worth mentioning that the literature mining provides a significant theoretical framework; additional practical validation or case studies would help to solidify the applicability and usefulness of the identified technology opportunities. Therefore, in this study, “gene editing technology in crop breeding” was selected as a case study for empirical research. The results of 13 technology combinations were based on the interpretation of domain experts and the resulting analysis. At the same time, domain experts were invited to evaluate 13 sets of technology combinations from three dimensions— scientificity, feasibility, and application prospects— to verify the effectiveness of our method.

Moreover, the three methods used in this study, namely, spaCy, SciBERT, and GTM, have cross- domain adaptability. By training specific domain data and adjusting parameters, it can be suitable for multi- domain data mining tasks. In the future, we will conduct empirical research in other fields to enhance the applicability of the methods.

### 4.3 Limitations and difficulties

This study quickly identified potential technology combination opportunities through literature mining methods, which avoids spending a lot of manpower to scientifically and effectively identify technology gaps. In our research, we utilized natural language processing, deep learning, and GTM to conduct an in-depth analysis of the literature on gene editing technology in crop breeding from the perspective of literature mining. The literature mining method can extract valuable research information from a large number of published works by scientists. Specific empirical research is often based on the application of one or several technologies in specific fields. Compared with specific empirical research, the method of literature content mining can provide researchers with more valuable information from a broader perspective, support their selection of specific methods and technologies, assist them in conducting specific empirical research, save their time and economic costs, and improve scientific research efficiency. It is worth noting that the results obtained from natural language processing methods still require the participation of domain experts and empirical research in specific fields to obtain more scientifically valuable results.

However, there are still some shortcomings: 1) the content differentiation of the identified technology combinations was not obvious enough, and there were multiple technical words appearing in multiple groups of technology combinations at the same time. 2) Only published academic literature was identified, and other data sources, such as invention patents and industry reports, were not involved. 3) The content analysis covered by the technical word combination and whether the technical combination can become a technological innovation opportunity for future development still needs expert judgment.

In the existing studies, researchers identify technological innovation opportunities from Science Citation Index (SCI) papers and Derwent patent data and select representative technical feature words by combining technical subject words and the Term Frequency-Inverse Document Frequency (TF-IDF) algorithm. Finally, potential technical subjects are evaluated by building a multi-dimensional feature index system and calculating the technological gap so as to realize the screening of technology opportunities (Yin et al., 2025; Li J. et al., 2025). The above research has achieved good experimental results, so it provides a reference for the in-depth research and optimization of this study. Specifically, it includes the following: 1) by obtaining keywords, calculating word TF-IDF values, and other methods, the importance of technical words is screened to obtain core technical words, thereby improving the differentiation between technical combinations; 2) expanding data sources (such as patents, policies, and reports), enriching the breadth of analysis, and improving the comprehensiveness of data coverage; and 3) by combining tools and methods such as artificial intelligence, semantic analysis, and technological innovation indicators, we can intelligently interpret and analyze the content of technology combinations, predict the possibility of technology combinations becoming opportunities for technological innovation in the future, and provide more reliable decision support for the future development of gene editing breeding.

## 5 Conclusions

In this study, through the comprehensive application of text mining technologies such as spaCy, Transformer, and GTM, from the perspective of literature data of gene editing technology in the field of crop breeding, the labeling of key technologies and the identification of technology combinations were carried out. The potential technological innovation opportunities of gene editing technology in the field of crop breeding were systematically mined and analyzed. The results showed that from the literature data from 2020 to 2024, 13 technology combinations were identified. Through interpretation and analysis, the content covers the multi-technology combination strategy of molecular genetic research, the core technology of gene function research in molecular genetics of biotic and abiotic stresses, the technical means of analyzing the molecular mechanisms of stress resistance, the technical scheme of genetic improvement, etc., which provides support for revealing the potential technological innovation opportunities of gene editing technology in the field of crop breeding. At the same time, the identification method of technological innovation opportunities proposed in this study can scientifically, objectively, and efficiently mine literature information and visually display the results in the form of technology map with technology combination, which provides reference significance for the identification of potential technological innovation opportunities in the field. However, there are still some shortcomings in this study: the content differentiation of identified technology combinations is not obvious enough, the sources of data mining are insufficient, and the analysis of technology combinations is not intelligent enough. In the future, we will continue to carry out in-depth research on the above issues to accelerate the application and technological innovation of gene editing technology in crop breeding.

## Data Availability

The original contributions presented in the study are included in the article/supplementary material. Further inquiries can be directed to the corresponding authors.
